# The contribution of sensory information asymmetry and bias of attribution to egocentric tendencies in effort comparison tasks

**DOI:** 10.3389/fpsyg.2024.1304372

**Published:** 2024-04-04

**Authors:** Caedyn Stinson, Igor Kagan, Arezoo Pooresmaeili

**Affiliations:** ^1^Perception and Cognition Lab, European Neuroscience Institute Göttingen—A Joint Initiative of the University Medical Center Göttingen and the Max Planck Society, Göttingen, Germany; ^2^Biological Psychology and Cognitive Neuroscience, Center for Cognitive Neuroscience Berlin, Department of Education and Psychology, Freie Universität Berlin, Berlin, Germany; ^3^Center for Adaptive Rationality, Max Planck Institute for Human Development, Berlin, Germany; ^4^Decision and Awareness Group, German Primate Center, Leibniz Institute for Primate Research, Göttingen, Germany; ^5^Leibniz Science Campus Primate Cognition, Göttingen, Germany; ^6^School of Psychology, University of Southampton, Southampton, United Kingdom

**Keywords:** effort perception, egocentric bias, social comparison, attribution bias, sensory availability

## Abstract

When comparing themselves with others, people often evaluate their own behaviors more favorably. This egocentric tendency is often categorized as a bias of attribution, with favorable self-evaluation resulting from differing explanations of one’s own behavior and that of others. However, studies on information availability in social contexts offer an alternative explanation, ascribing egocentric biases to the inherent informational asymmetries between performing an action and merely observing it. Since biases of attribution and availability often co-exist and interact with each other, it is not known whether they are both necessary for the egocentric biases to emerge. In this study, we used a design that allowed us to directly compare the contribution of these two distinct sources of bias to judgements about the difficulty of an effortful task. Participants exhibited no attribution bias as judgements made for themselves did not differ from those made for others. Importantly, however, participants perceived the tasks they actively performed to be harder than the tasks they observed, and this bias was magnified as the overall task difficulty increased. These findings suggest that information asymmetries inherent to the difference between actively performing a task and observing it can drive egocentric biases in effort evaluations on their own and without a contribution from biases of attribution.

## 1 Introduction

Social comparison between self and others is a fundamental mechanism that influences various types of human judgement (Festinger, 1954; Buunk and Mussweiler, 2001; Crusius et al., 2022). It often comes into play in cognitive processes that require the integration of social information, such as using peers as reference points to evaluate one’s own abilities (Dijkstra et al., 2008; Wolff et al., 2018; El Zein et al., 2019), considering and evaluating opinions (Festinger, 1954; Buunk and Gibbons, 2007), incorporating the actions of others into the learning process (Mattar and Gribble, 2005; Dijkstra et al., 2008), and conforming to social norms (Allcott, 2011; Yun and Silk, 2011). Interestingly, social comparison is also evident when there is no explicit need to integrate social information, such as when people evaluate their reward outcomes and exerted effort. For instance, the value a person attaches to a reward depends not just on the reward’s absolute value but also its value relative to that received by their peers (Fliessbach et al., 2007; Boyce et al., 2010; Card et al., 2012; Zell et al., 2019). People also monitor relative effort across their social group in order to identify social loafing – the tendency to spend less effort when one is judged as part of a group- (Karau and Williams, 1994) and to ensure that reward is meted out proportionately to effort (Akerlof and Yellen, 1990; Bland et al., 2017).

Despite their ubiquity, comparisons between oneself and others are subject to a range of biases (for a review see Fiske and Taylor, 2016). Typically, these biases are in the direction of favorable self-assessment and as such are widely referred to as *egocentric* or *self-serving biases* (for a review see Greenberg et al., 1982; Blaine and Crocker, 1993). Egocentric biases include the overestimation of one’s contribution to cooperative tasks (Ross and Sicoly, 1979; Kruger and Dunning, 1999; Corgnet and Gunia, 2010); the better-than-average effect, in which more than 50% of people consider themselves to be above average in a given task (Zell et al., 2019); the willingness of feuding parties to pursue economically irrational legal actions (Loewenstein et al., 1993); and overestimation of one’s own impact on outcomes (Berberian et al., 2012). Unfavorable biases in social comparison have also been demonstrated (Davidai and Deri, 2019), with people displaying a tendency to compare upwards when evaluating social standing (Lup et al., 2015) and wages (Harris et al., 2008), leading to dissatisfaction with one’s own situation.

Biases in social comparison have been extensively studied in the field of attribution theory (Weiner, 1985; Weiner et al., 1987). Attribution theory posits that biases in social comparison arise from asymmetrical attribution in people’s causal explanations of behavior (for a review see Fiske and Taylor, 2016). According to the theory, explanatory sources are asymmetrically attributed for self and others to either external (i.e., situational) or internal (i.e., dispositional stemming from the actor) causes (Jones and Nisbett, 1987; Gilbert and Malone, 1995; Pronin, 2008). For instance, an important meta-analysis (Malle, 2006) found that people were more likely to attribute their own success to internal factors and their failures to external factors.

While studies on attribution theory provide a wealth of evidence of social comparison asymmetries, they do not offer a causal explanation of the underlying mechanisms. Studies that investigate the information available to individuals, however, offer more insight into the matter. These studies suggest that asymmetries in social comparison may be rooted in the fact that a person’s own intentions and the direct sensory input from their actions are simply more available to them than are the intentions and sensory input of others (Pronin, 2008). This availability-driven bias is evident in the overestimation of one’s own contributions to group tasks (Ross and Sicoly, 1979), the underestimation of collaborators in group tasks with increased physical distance (Corgnet and Gunia, 2010), and the greater saliency of external factors that impede people from attaining their goals compared to factors that assist them (Davidai and Gilovich, 2016).

Attribution and availability biases have been associated with specific functions, such as motivational or cognitive roles (Shepperd et al., 2008; for reviews see Fiske and Taylor, 2016). The motivational role finds support in studies indicating that egocentric biases enhance self-perception (Shepperd et al., 2008; Fiske and Taylor, 2016; Sedikides and Alicke, 2019), hence being magnified when maintaining enhanced self-views is critical (Sedikides et al., 1998; Lammers and Burgmer, 2019). The cognitive role of egocentric biases is underscored by the observation that humans have much more uncertainty regarding the mental states and contribution of others compared to themselves (Chambers and Windschitl, 2004; Pronin et al., 2004; Kruger et al., 2008). Increasing uncertainty, for instance, by rendering trait definitions ambiguous (Dunning et al., 1989) or making task outcomes variable (Rollwage et al., 2020), amplifies the egocentric bias. However, both cognitive and motivational processes jointly contribute to social comparison biases, presenting challenges in determining their relative impacts (Shepperd et al., 2008).

Another approach taken by the previous studies has been to specify the distinct type of the asymmetries which leads to biases in social comparison. For instance, explanations varying from an asymmetry between *self* and *other* (e.g., Weiner, 1985; Pronin, 2008), or *active* and *observe* (e.g., Jones and Nisbett, 1987; Malle, 2006) in attribution or availability theories, respectively, have been put forward to explain egocentric biases. These asymmetries are intuitive, do in fact match the everyday experience and can be experimentally manipulated.

Asymmetries between self and other (self/other asymmetry) arise when people process sensory information about themselves and others. An individual exhibiting a self/other bias would receive information without an inherent bias. But they would interpret this information in a biased manner. For example, when considering contributions to household tasks, a person may receive equal amounts of information about their own and their partner’s efforts. But their preconceived representations (e.g., “my partner is typically lazy”/“I normally get housework done quickly”) would lead them to an asymmetric consideration of this information, and result in a biased perception.

Asymmetries between being active or observing (active/observe asymmetry), in contrast, are rooted in the disparity of available information from a first-person perspective. An individual exhibiting an active/observe bias would receive more information about their own contribution than about their partner’s contribution. This asymmetry of information would lead to the biased perception that they contribute more to the housework.

The distinction between self/other and active/observe asymmetries is important for understanding the underlying mechanisms of egocentric biases. Recognizing that self/other biases arise from biased interpretations of unbiased sensory inputs, while active/observe biases arise from asymmetric sensory input interpreted in an unbiased manner, can help delineate the neurocognitive pathways involved. It emphasizes the stages at which biases can occur – the self/other bias in the recall or representation of information, and the active/observe bias in the information that is initially encoded.

Despite their importance, it is unknown whether self/other (i.e., attribution) and active/observe (i.e., availability) asymmetries are both necessary for the egocentric biases to emerge and what psychophysiological mechanisms underlie them. Importantly, the two sources of bias often co-exist (e.g., we may regard ourselves to be more industrious than our partner and at the same time underestimate their contribution to a task due to the lack of information regarding how much they worked), and they could potentially interact (such as when a favorable self-view leads agents to preferentially process the self-related information; Kunda, 1987). These features present a dilemma for an experimental paradigm which seeks to isolate and distinctively analyze the role of each factor in egocentric biases.

**Figure 1:**
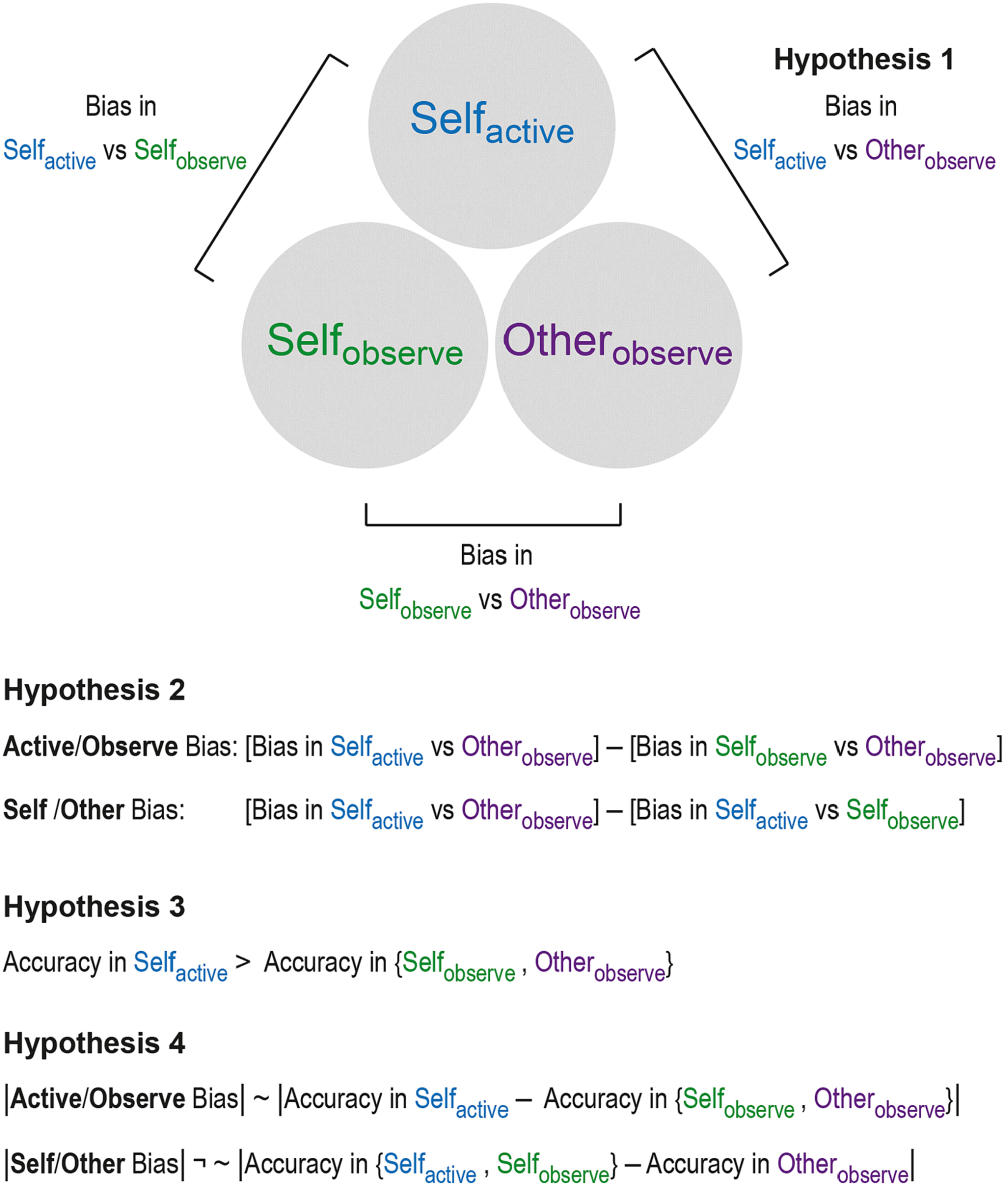
Design and hypotheses of the study. Three conditions were considered: participants assessed the difficulty of a sensorimotor task (see Figure 2 for the illustration of the task) when they either *actively* performed it (*Self_active_*) or when they *observed* it being done: by another person (*Other_observe_*) or by themselves (*Self_observe_*). In the latter two conditions (*Other_observe_* and *Self_observe_*), prerecorded tasks from a previous session when the participants did the task themselves (i.e., *Self_active_*) were played back to them. This design allowed us to test the key hypotheses of the study. Hypothesis 1 posited that participants’ ratings of the task difficulty will significantly differ between *Self_active_* and *Other_observe_* trials (i.e., *Self_active_ – Other_observe_* ≠ 0). Hypothesis 2 held that both an active/observe and a self/other bias will be detected. Here, the information provided by *Self_observe_* trials is used to determine whether a putative bias observed in Hypothesis 1 is due to an active/observe or a self/other bias. To do so, in each case the bias (*Self_active_ – Other_observe_*) is corrected for the differences arising in comparisons with the *Self_observe_* condition. Hypothesis 3 posited that accuracies will be higher in *active* compared to *observe* conditions, as participants have more access to sensorimotor information when they actively perform the task. Finally, Hypothesis 4 posited that the accuracy difference detected by Hypothesis 3 is only correlated with the active/observe and not the self/other bias.

In the present study, we aimed to dissociate the two potential sources of egocentric bias—self/other asymmetry (attribution) and active/observe asymmetry (availability). To do so, we employed a paradigm previously used to test how the external information (reward feedback) is integrated with the internal (sensorimotor) representations of physical effort when effort judgements are made for self (Pooresmaeili et al., 2015) or for others (Rollwage et al., 2020). To focus on the social comparisons during judgements of effort, here we removed the reward information from the tasks used in these previous studies and instead had participants directly compare the difficulty of the task when it was performed by themselves or a partner. To account for the information disparity between the active performance of a task by oneself (referred to as Self_active_, see Figure 1) and merely observing it being done by a partner (referred to as Other_observe_, see Figure 1), we added a condition in which participants also watched their own prerecorded performance of the task (referred to as Self_observe_, see Figure 1). This critical control condition, which to the best of our knowledge has never been considered in the previous studies, was the key factor in deciding whether egocentric biases stem from a self-favoring attribution or are instead due to the impoverished information available to the agents while they observe a task being done.

Using this design, we hypothesized that we would detect Self_active_ vs. Other_observe_ bias, as well as both *self/other* and *active/observe* biases at the group level (Hypothesis 1 and 2, see Methods and Figure 1). The *self/other* bias was expected to manifest as a higher rating of the task difficulty when the task was done by the participants themselves as opposed to a partner. This is because a higher difficulty level would imply that participants had to exert a higher amount of effort and by doing so succeed in a more challenging situation compared to their peers, both of which – i.e., higher effort expenditure and success – are socially desirable traits (Miller and Ross, 1975). Since the task we employed was an effortful sensorimotor task, it was also expected that participants would rate the difficulty higher when they actively performed the task compared to when they merely observed it being done by themselves or by others (i.e., *active/observe* bias). Due to more sensorimotor and introspective information being available when a task is actively performed, we also predicted that participants would exhibit greater accuracy in *active* compared to *observe* trials (Hypothesis 3), and that the accuracy disparity between *active* and *observe* conditions would correlate with the degree of *active/observe* bias, but not with *self/other* bias (Hypothesis 4).

## 2 Methods

### 2.1 Participants

We report all data exclusions, all manipulations, and all measures in this study. The study was approved by the Freie Universität Berlin Department of Educational Science and Psychology Ethics Commission. A preregistered power analysis^1^ using data from Rollwage et al. (2020) indicated that a sample size of 49 was required. To account for participant dropout or exclusion, 63 participants were recruited, with 51 adults aged 18–35 years old included in the final analysis (31 female, 20 male; mean age 24.4; SD = 4.1 years; 45 right-handed). Of the 12 individuals who participated in the study but were excluded from analysis, 10 were excluded for not meeting the pre-registered accuracy threshold of >80% correct on trials where the easiest task was compared with the most difficult, one was excluded for correctly identifying the experimental manipulation in a post-experimental survey, and one was excluded because their performance fell 4.20 standard deviations from the sample mean.

**Figure 2:**
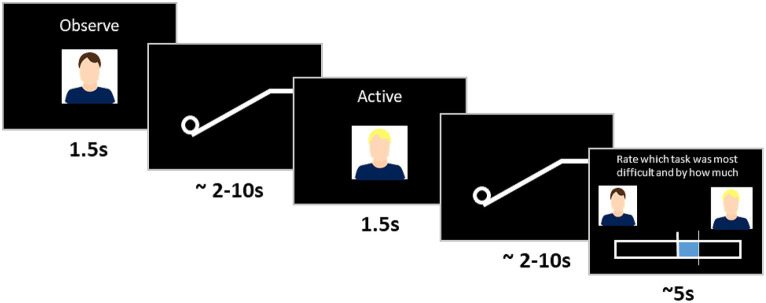
Two-interval forced choice workflow. Each trial comprised two tasks (pushing a ball, or watching a ball be pushed up a ramp), performed by participants themselves (self condition) or someone else (other condition). Before each task, a photo of the person who performed the task and the instruction whether the participant had to perform the task (active condition) or observe it being performed (observe condition) was displayed onscreen. After the two tasks were completed, participants rated which was more difficult.

Participants were recruited from Freie Universität Berlin via mailing lists and on-campus advertising and from social media groups for English speakers living in Berlin. Participants were required to have normal/corrected to normal vision, no current health problems, no history of psychiatric or neurological disorders (self-reported), and sufficient English skills to understand the task instructions and the consent documents. Participants received a base payment of €8/h (the experiment took 4 h) plus bonus payments to encourage performance and attention: extra money was offered for completing *active* tasks and for correctly detecting ball color changes on selected trials (see Figure 2 and section 2.3.1 for the description of this task). Participants were also rewarded for correctly identifying the more difficult of the two tasks in each trial, in order to encourage the accuracy of the judgements. The maximum payment for the complete study was €45.34. Students of Freie Universität Berlin could opt to receive course credits in lieu of the base payment, but still received any extra money they earned, to ensure motivation was consistent across all participants. These students received their overall winnings without the hourly pay, for a maximum of €13.34. Of the 55 participants who completed the full study (four of whom were excluded), 30 were fully paid, receiving a mean payment of €42.24, and 25 received four course credit hours and a mean payment of €8.94.

### 2.2 Apparatus and stimuli

The experimental stimuli was produced using MATLAB (2016a, The MathWorks, Inc., Natick, Massachusetts, United States) and Psychophysics Toolbox (Brainard, 1997; Pelli, 1997). Four desktop PCs were used [6GB RAM; Intel® Core(TM) 2 Duo Processors E8400] with Samsung SyncMaster monitors (model: 943BR; screen width 29.4 cm; screen height 16.6 cm; resolution 1280×1024 pixels), and Dell keyboards (model: E145614). The screen was placed 50 cm from the estimated viewing position. The computers were positioned so that participants were unable to see the other participants’ screens.

The task featured a white ball (radius = 30 pixels), and a ramp that culminated in an upper plateau (ramp length = 550 pixels, angle = 30°, plateau length = 150 pixels; Figure 2) with a black background. Participants moved the ball up the ramp by pressing left and right keys in alternating order and were instructed to use the index and middle fingers of their dominant hand for the entire experiment. Each key press resulted in a constant amount of displacement of 25 pixels up the ramp. Participants wore over-ear high ambient noise-attenuating headphones (Sennheiser HD 280/380 Pro), which delivered a beep for every key press. The ball’s progress was opposed by a “gravity level” that rolled the ball down the ramp at a constant rate. If participants stopped pressing keys, or pressed too slowly, the ball would roll back toward the start point, where it would remain until key pressing resumed. A task consisted of either actively moving the ball (*active* condition) or watching the ball being moved (*observe* condition). Each trial consisted of two tasks. After each trial participants rated the relative task difficulty of the two tasks using a rating bar (1,024 pixels wide × 102 pixels high) and rating slider (10 pixels wide × 204 pixels tall).

### 2.3 Experimental design

#### 2.3.1 Paradigm overview

The ball and ramp task had three conditions (Figure 1):

- *self_active_*: Participants used the keyboard to move the ball up the ramp.
- *other_observe:_* Participants watched a recording of what they were told was their partner moving the ball up the ramp (but was in fact a recording of their own performance).
- *self_observe_*: Participants watched a recording of themselves moving the ball up the ramp.

The experiment was conducted as a two-interval forced choice paradigm. A two-interval forced choice consists of two tasks being presented sequentially, with participants then choosing between the two tasks – in this case indicating which they thought was more difficult (i.e., which had the greater gravitational force) and by how much. As this was a forced choice paradigm, participants had to select one of the two items and were unable to register the tasks as equally difficult. This continuous rating schema provided both binary data (which task was more difficult) and continuous data (the first task was *x* more/less difficult than the second task). A cover task where the ball would briefly change color on one of the two tasks was also included so that participants did not focus on guessing the purpose of the experiment. On 15% of trials, participants were required to identify the task in which the ball changed color.

The two-interval forced choice trials were performed between conditions (self_active_ vs. other_observe_, self_active_ vs. self_observe_, self_observe_ vs. other_observe_) to calculate bias, and within each condition (self_active_ vs. self_active_, other_observe_ vs. other_observe_, self_observe_ vs. self_observe_) to calculate accuracy. We utilized within-subjects design, in which all participants performed all conditions. Each participant was paired with a gender-matched partner who was not known to them before the study. To reinforce the social nature of the task, a photo of the participant performing the task accompanied by the word “active” or “observe” was shown above the rating bar before each task (Figure 2). To prevent confounds arising from differences in participant performance, participants were told that they were observing their partner’s prerecorded activity, when in fact both self_observe_ and other_observe_ conditions were their own prerecorded activity. All participants completed the study over two separate days. They completed the first day of the study concurrently with their partner, but to prevent co-ordination issues, performed the second day of the study separately, between 1 and 8 days later (mean = 3.17 days later). The study consisted of five sections: maximum effort estimation, practice trials, prerecording trials, between-condition main experiment, and within-condition main experiment (the order of between- and within-conditions was counterbalanced across participants).

#### 2.3.2 Maximum effort estimation

The first section, maximum effort estimation, consisted of three sequential 10-s trials with a gravity force that increased exponentially as the ball moved up the ramp. To encourage participants to exert maximum effort a bonus reward of €1 was offered for reaching the top of the ramp (no participant reached the top). The values for each participant’s gravity levels were calculated from these trials by measuring the location of the 80th percentile of the ball’s lateral displacement for each of the three trials, excluding the trial with the lowest value, then averaging the remaining two values and calculating the gravity (*g_max_*) opposing the ball at this point. That is, *g_max_* was the maximum gravity level participants could resist for two seconds in two out of three trials. For the experiment, six gravity levels were used, each calculated as a percentage of *g_max_* (Table 1). Participants were informed that there were different levels of difficulty but were not told how many.

**Table 1:** Gravity levels as determined by g_max_.

Gravity level	% of *g_max_*
1	20%
2	26%
3	32%
4	38%
5	44%
6	50%

This table gives the difficulty level of each gravity level in the experiment as a fraction of the maximum gravity level. The maximum gravity level (*g*_max_) is determined by the highest gravity level participants could resist for 2 s in two out of three trials, where their maximum effort was measured.

#### 2.3.3 Practice trials

Participants performed six practice trials to become familiar with the task, comparing *self_active_* gravity levels of 1v6, 6v1, 1v4, 5v2, and 3v1. During these trials, participants received feedback on their choices to ensure that they understood the paradigm. At no other point during the experiment was feedback given. The gravity differences between the two tasks (Δ*grav*) for these trials were kept intentionally large to prevent participants from becoming artificially proficient in the *self_active_* condition. Participants could repeat the practice section if they wished.

#### 2.3.4 Prerecorded trials

Next, participants performed the *self_active_* tasks which would be recorded and played back to them during the main experiment (unbeknownst to them) as both the *self_observe_* and *other_observe_* conditions. The tasks were presented as two-interval forced choice trials. Participants’ performances were recorded by logging the timing of their correctly ordered alternate left and right key presses. This made it possible to show replays of the participants’ pre-recorded tasks within 0.01 s of accuracy, without the need for video recording.

Our pilot studies showed that participants were more fatigued when performing the within condition *self_active vs_ self_active_* trials, resulting in slower completion of each task compared to between condition trials between *self_active_* and the two observe conditions. To ensure that the tasks used for the *observe* condition matched the fatigue levels of the *active* tasks, we prerecorded tasks for the within-condition main experiment from *self_active vs_ self_active_* trials and tasks for the between-condition main experiment from *self_active vs_ self_observe_* trials. Participants were instructed to take breaks if they felt fatigued during the prerecording stage and were encouraged to move the ball up the ramp as fast as they could. Prerecorded trials were excluded if participants paused for more than 3 s between key presses in order to eliminate easily identifiable *observe* tasks where the ball rolled back extensively.

Participants were told they were observing a partner performing the *other_observe_* tasks but were actually watching their own prerecorded activity. This prevented potential confounds arising from differences in participant ability. To ensure that participants believed they were observing their partner, they had to wait until both they and their partner had finished the prerecording section of the experiment, at which point the experimenter manually transferred files between the two computers using a USB stick. We assessed whether participants believed they were observing a partner’s trials in a post-experiment interview in which they were asked, “How do you think your partner compared to you in ability? Were they better, worse or the same?” Participants were then informed that they had seen their own prerecorded trials for the *other_observe_* condition and were asked, “On a scale of 1–10, with 1 being not at all, and 10 being completely, to what degree did you suspect the other participant’s activity wasn’t their own?” If participants indicated any skepticism after the first question, or if they answered the follow-up question with a rating of 10, their data was removed from the analysis. Only one participant was removed after identifying the manipulation in response to the first question. No participants indicated above an ‘8’ for the second question (full results in Supplementary Figure S5).

#### 2.3.5 Main experiment: between-condition two-interval forced choices

The between-condition and within-condition portions of the study were performed on different days, with the order counterbalanced across participants. To measure self/other and active/observe bias participants performed forced choice trials comparing *self_active_* vs. *other_observe_*, *self_observe_* vs. *other_observe_*, and *self_active_* vs. *self_observe_*. Each trial was categorized by the difference in gravity level between the two tasks (Δ*grav*). For between-condition trials, Δ*grav* was given by $\Delta grav=glevel_{i}-glevel_{j}$ , where $glevel_{i}$ and $glevel_{j}$ are the gravity level of conditions *i* and *j*, respectively. Six steps were used: Δ*grav* = −5, −3, −1, +1, +3, +5. These comprised of comparisons of the following gravity levels (italicized): ±1: *1v2, 2v3, 3v4, 4v5, 5v6*; ±3: *1v4, 2v5, 3v6*; ±5: *1v6*. Twenty trials were performed for each Δgrav for a total of 120 trials per condition, or 360 trials altogether. Trials were presented in blocks of 30. The blocks were presented in pseudorandomized order: Each set of three blocks comprised one block from each of the three comparisons presented in a randomized order. The Δ*grav* values and their associated gravity levels were balanced across blocks and presented in random order.

#### 2.3.6 Main experiment: within-condition two-interval forced choices

Within-condition two-interval forced choices allowed us to compare participant accuracy in one *active* and two *observe* conditions. For these trials, Δ*grav* was given by $\Delta grav=\;|glevel_{1}-glevel_{2}|$ , where $glevel_{1}$ and $glevel_{2}$ are the gravity levels of the first and second task in each trial. Five Δ*grav* levels (1–5) were composed of the following (gravity levels italicised), respectively: 1: *1v2, 2v3, 3v4, 4v5, 5v6*; 2: *1v3, 2v4, 3v5, 4v6*; 3: *1v4, 2v5, 3v6*; 4: *1v5, 2v6*; 5: *1v6*. Each Δgrav level was appeared in 20 trials for a total of 100 trials per condition, which were performed in four blocks of 25 trials. These four blocks for each of the three conditions were pseudorandomized as in the between-condition main experiment.

### 2.4 Analysis and hypotheses

All analyses were performed using MATLAB (2016a, The MathWorks, Inc., Natick, Massachusetts, United States). The following analysis and hypotheses (see also Figure 1) were preregistered at https://osf.io/6pwvf/?view_only=fcbbd52a25ca44e9989254a97748868d. Exploratory analysis was also performed and is described in a separate section (2.4.5) for clarity.

#### 2.4.1 Hypothesis 1: bias in self_active_ vs. other_observe_ comparisons

We hypothesized that participants would exhibit a bias when comparing the perceived difficulty of tasks they actively performed with tasks they observed their partner performing. This was measured by fitting the binary rating data from all *self_active_* vs. *other_observe_* trials to a psychometric function with the Δ*grav* level for each trial as the independent variable and the proportion of trials rated as easier for *other_observe_* as the dependent variable, and assessing the point of subjective equality (PSE). Fitting of the psychometric functions was done using the MATLAB function psignfit (Schütt et al., 2016). The PSE for *self_active_* vs. *other_observe_* was calculated from the psychometric function of each individual (for an example of the actual psychometric curves in one example participant see Supplementary Figure S1). Hypothesis 1 was assessed, against the null hypothesis that PSE for *self_active_* vs. *other_observe_* trials did not differ, using a two-tailed one sample *t*-test with significance set at *p* < 0.05.

#### 2.4.2 Hypothesis 2: biases for active/observe and self/other

We hypothesized that two dissociable biases would be detected: one driven by asymmetric sensory information on *active* vs. *observe* trials, and another driven by attributional differences on *self* vs. *other* trials (Figure 1). This was predicted to hold even if Hypothesis 1 showed no net bias—in that case, the biases would still be present, but would offset each other.

We dissociated the potential sources of bias—active/observe asymmetry and self/other asymmetry—by calculating the relative PSE between the conditions. The active/observe bias (*PSE_active vs observe_*) was calculated as the difference in PSE of the *self_active_* vs. *other_observe_* and the *self_observe_* vs. *other_observe_* conditions (Equation 1), as this comparison isolated the bias attributed to sensory differences when comparing own task difficulty with that of a partner from those related to the differences between self and other (i.e., *self_observe_* vs. *other_observe_*). Similarly, the self/other bias (*PSE_self vs other_*) was calculated as the difference in PSE between *self_active_* vs. *other_observe_* and *self_active_* vs. *self_observe_* (Equation 2). This allowed us to isolate the bias stemming from attributional differences, controlling for the differences stemming from active/observe asymmetry (*self_active_* vs. *self_observe_*).


(1) $$\begin{array}{l} PSE_{active\;vs\;observe}=PSE\left(self_{active}\;vs\;other_{observe}\right)\\ -PSE\left(self_{observe}\;vs\;other_{observe}\right) \end{array}$$

(2) $$\begin{array}{l} PSE_{self\;vs\;other}=PSE\left(self_{active}\;vs\;other_{observe}\right)\\ -PSE\left(self_{active}\;vs\;self_{observe}\right) \end{array}$$

For each participant, PSEs were measured for the three between-condition comparisons (*self_active_* vs. *other_observe_*, *self_observe_* vs. *other_observe_*, and *self_active_* vs. *self_observe_*) from the psychometrics (same fitting procedure as in Hypothesis 1) with the Δ*grav* as the independent variable and the proportion of trials rated as easier for Condition 2 in each comparison as the dependent variable. Conditions 1 and 2 were defined as shown in Table 2.

**Table 2:** Definitions of conditions 1 and 2 for psychometric between-condition analysis.

2IFC comparison	Condition 1	Condition 2
*self_active_ vs other_observe_*	*self_active_*	*other_observe_*
*self_observe_ vs other_observe_*	*self_observe_*	*other_observe_*
*self_active_ vs self_observe_*	*self_active_*	*self_observe_*

This table indicates conditions 1 and 2 for each two-interval forced choice (2IFC) comparison. As the main analysis of interest was *self_active_* vs *other_observe_*, *self_active_* was set as Condition 1 and *other_observe_* was set as Condition 2. Because *self_observe_* was used as a control condition to extract the two biases of interest (self vs other, active vs observe), it replaced *self_active_* in the second comparison and *other_observe_* in the third comparison.

The presence of bias for active/observe and self/observe was analyzed using a linear mixed effects model (MATLAB function fitlme; Pinheiro and Bates, 1996). Values for *PSE(self_active_* vs. *other_observe_)*, *PSE(self_active_* vs. *self_observe_),* and *PSE(self_observe_* vs. *other_observe_)* were entered as dependent variables, with categorical parameters indicating whether the trial involved a self/other or active/observe asymmetry added as fixed effects parameters. Random effects were added to allow for random intercepts for each participant as well as for the two sources of bias. To test Hypothesis 2, the full linear mixed effects model was compared to reduced models with fixed effects for only s*elf* vs. *other*, *active* vs. *observe* or neither (*null*) using likelihood ratio testing (MATLAB function compare (lme, altlme); Hox and Maas, 2005). Hypothesis 2 was also analyzed using a two-tailed one sample *t*-test (α = 0.05) to determine whether *PSE_self vs other_* and *PSE_active vs observe_* were significantly different from zero.

#### 2.4.3 Hypothesis 3: greater accuracy in active tasks relative to observe tasks

We hypothesized that participants would be more accurate in their ratings when they had performed the effortful task than when they had observed it. As a proxy for accuracy, we measured the participant’s just-noticeable-difference (JND) – in this case the minimum gravity difference between two tasks needed for a participant to accurately determine which task was more difficult 80% of the time.

Statistical comparison of accuracy in *self_active_*, *other_observe_*, and *self_observe_* conditions was performed using a repeated measures analysis of variance (ANOVA). Multiple comparisons with a Bonferroni correction were used to provide statistical tests of significance between each of the three conditions.

#### 2.4.4 Hypothesis 4: do differences in accuracy correlate with bias

We hypothesized that the level of active/observe bias would correlate with greater accuracy differences between *active* and *observe* trials, but the self/other bias would not correlate with accuracy differences between *self* and *other* trials. Theoretical considerations mentioned in the introduction and the pilot data suggested that bias between *active* and *observe* tasks may be driven by asymmetries in accuracy when either performing or observing a task. To explore this, a Pearson’s correlation (MATLAB function corrcoef) was performed between participants’ *PSE_active vs observe_* and the JND between the *active* and *observe* conditions, as given by Equation 3, where *PSE_active vs observe_* is the bias between *active* and *observe* trials (see 2.4.2 Hypothesis 2 2) and the right side of the equation captures the accuracy difference between the *observe* within-condition trials and the *active* within-condition trials.


(3) $$\begin{array}{l} |PSE_{active\;vs\;observe}|\sim |((JND_{self+observe}+\\ JND_{other+observe})/2–JND_{self+active}| \end{array}$$

The absolute value of both sides of the equation is taken under the assumption that the accuracy difference, but not the direction of the difference, drives the magnitude of the active/observe bias. The statistical threshold for significance was set at *p* < 0.05.

The same analysis was also performed for *PSE_self vs other_* (Equation 4), with the expectation that this relationship would not be correlated, as participants would not receive any information which would increase their accuracy in either the *self* or *other* conditions.


(4) $$\begin{array}{l} \left|PSE_{self\;vs\;other}|\sim |((JND_{self+active}+JND_{self+observe}\right)\\ /2–JND_{other+observe})| \end{array}$$

#### 2.4.5 Exploratory analysis

The preregistered analysis focused solely on the binary two-interval forced choice decision data. However, continuous rating data was also recorded for each trial by measuring the displacement of the ratings bar. Therefore, to complement the psychometric modelling of the preregistered analysis, the analysis of Hypotheses 1 and 2 was repeated using the continuous rating data from the between-condition comparisons. Using the continuous rating data made it possible to capture subtleties in perceptual judgement that the binary data may miss.

We also used the continuous rating data to analyze the effect of task difficulty on active/observe bias. If bias in social comparison was driven by sensory asymmetries, the perceived bias would be expected to increase as task difficulty increased, since with an increase in task difficulty the disparity between actively doing the task and merely observing it would grow. To test this, bias was calculated for each condition for trials where multiple combinations of gravity levels were used to form the same *Δgrav* (i.e., trials where Δ*grav* = +1, −1, +3, and − 3). As Δ*grav* = ±5 was only possible in one combination (6 vs. 1 and 1 vs. 6), it was not used in this analysis. For each permutation, we tested active/observe and self/other bias for significant deviation from zero using two-sided one sample t-tests. Furthermore, a repeated measures ANOVA assessed whether there was a significant interaction between relative task difficulty and active/observe bias for each of the four Δgrav levels. Only the between-condition trials where unique permutations for each Δ*grav* levels occurred (Δ*grav* = +3, +1, −1, −3) were included in the ANOVA. Significant effects in ANOVA were followed up by *post hoc* pair-wise comparisons (MATLAB function multcompare, with Bonferroni correction).

Finally, based on results from this exploratory analysis of task difficulty, a linear mixed effects model was run using the continuous rating data from all trials in order to determine whether task difficulty was driving the level of active/observe bias. The model included fixed effects parameters indicating whether the trial involved a self/other asymmetry, an active/observe asymmetry, the Δ*grav* for each trial, the condition presented first (to capture variance in how participants were influenced by presentation order), and the gravity level of Condition 1 for each trial (to capture the effect of overall task difficulty). As task difficulty was expected to drive the amount of bias ascribed to the active/observe asymmetry, the fixed effects parameter for the gravity level of Condition 1 was included as an interaction term with the parameter for active/observe asymmetry. The gravity level of Condition 1 was also included as an interaction term with the parameter indicating which condition was presented first. As random effects, we included random intercepts for each participant, and by-participant random slopes for each fixed effect. We performed nested model comparisons with reduced versions of this full model to evaluate model performance. As with all previous model comparisons, ΔBIC (Bayesian information criteria; Schwarz, 1978) and ΔAIC (Akaike’s information criteria; Burnham and Anderson, 1998) were performed; a theoretical likelihood test (Hox and Maas, 2005) was used in the event of disagreement between these two criteria.

## 3 Results

### 3.1 Hypothesis 1: no bias between *self_active_* and *other_observe_*

**Figure 3:**
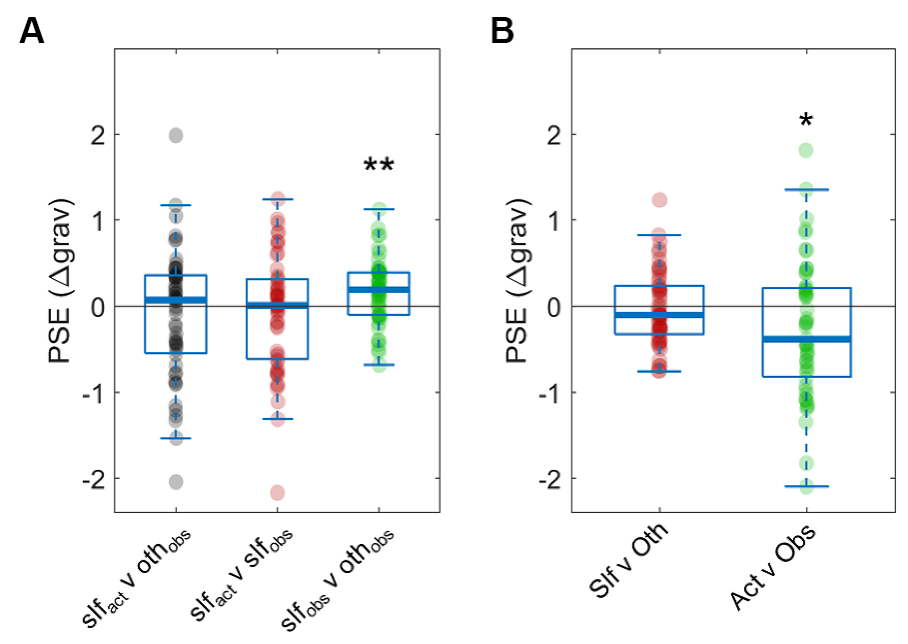
Point of subjective equality (PSE) between conditions. Both panels plot the PSE between conditions as calculated by psychometric functions, with negative values indicating an overestimation of the difficulty of the condition listed first (i.e., Condition 1). **(A)** Two-interval forced choice results between the three conditions: performing the task (slf_act_), watching a partner perform the task (oth_obs_), and watching a recording of oneself performing the task (slf_obs_). **(B)** Attribution bias (Slf vs. Oth) and sensory asymmetry bias (Act vs. Obs) for all participants. *p ≤ 0.05, **p ≤ 0.01. Note that in the plots the thick horizontal lines denote the median of the distribution.

To test Hypothesis 1, we inspected PSEs between conditions (Figure 3A). No bias between *self_active_* and *other_observe_* was detected at the group level [*Mean* = 0.106, *SD* = 0.75, *t*(50) = −1.01, *p* = 0.3166, *d* = 0.1416]. Participants exhibited PSEs that ranged from underestimating (Figure 3A, positive values) to overestimating (Figure 3A, negative values) the difficulty of tasks they performed themselves. However, as there was no systematic bias across all participants, we were unable to reject the null hypothesis. Analysis using the continuous rating data also found no significant bias [*Mean* = −0.1066, *SD* = 0.5732, *t*(50) = −1.33, *p* = 0.1904, *d* = 0.1860] (Supplementary Figure S2A).

It is worth noting that no bias was detected also in the *self_active_* vs. *self_observe_* condition [*Mean* = −0.0797, *SD* = 0.68, *t*(50) = −0.841, *p* = 0.4044, *d* = 0.1177; Figure 3A]. However, participants significantly underestimated the difficulty of their own trials in *self_observe_* vs. *other_observe_* comparisons [*Mean* = 0.168, *SD* = 0.3762, *t*(50) = 3.19, *p* = 0.0024, *d* = 0.4472], even though these trials compared identical stimuli. This result may indicate evidence of metacognitive processes, where participants consciously accounted for a potential egocentric bias due to the comparative nature of the task (we address this possibility further in the Discussion section).

### 3.2 Hypothesis 2: active/observe bias detected, but no bias for self/other

Participants exhibited no self/other bias as a group [*Mean* = −0.0267, *SD* = 0.4366, *t*(50) = −0.4360, *p* = 0.6647, *d* = 0.037; Figure 3B]. However, a significant active/observe bias emerged, with participants overestimating the difficulty of tasks they performed themselves [*Mean* = −0. 2,746, *SD* = 0.7859, *t*(50) = −2.746, *p* = 0.0159, *d* = 0.462]. Hypothesis 2 stated that both biases would be detected; however the null hypothesis was only rejected for one type of bias (active/observe). Therefore, results point toward a single source of bias for participants in this paradigm, driven by the asymmetries of performing or observing the task.

The mixed effects modeling mirrored this finding, with AIC and BIC model selection choosing the Active vs. Observe and Null model, respectively (Table 3), while a likelihood ratio test suggested that the Active vs. Observe model explained the data significantly better [χ^2^(1) = 4.4984, *p* = 0.0332]. In the Active vs. Observe model, performing the task increased perceived difficulty by 0.261 ± 0.082 (standard errors) gravity levels. However, in the analysis using continuous ratings, both AIC and BIC model comparisons selected the Active vs. Observe model. This finding was further supported by a likelihood ratio test: χ^2^(1) = 46.15, *p* < 0.0001 (Supplementary Table S1).

**Table 3:** Attribution bias and sensory asymmetry bias model comparison.

Model	ΔAIC	ΔBIC	Log likelihood	*R*^2^ (adjusted)
Full	1.92	5.44	−132.41	0.3789
Active vs. Observe	0	0.49	−132.45	0.3823
Self vs. Other	4.53	5.02	−134.72	0.3714
Null	2.53	0	−134.72	0.3758

Mixed-effect models featuring fixed effects for both attribution bias and sensory asymmetry bias (Full) or only one bias (Self vs Other, Active vs Observe) were fit with the between-condition point of subjective equality (PSE) data.

### 3.3 Hypothesis 3: greater accuracy in active tasks than observe tasks

**Figure 4:**
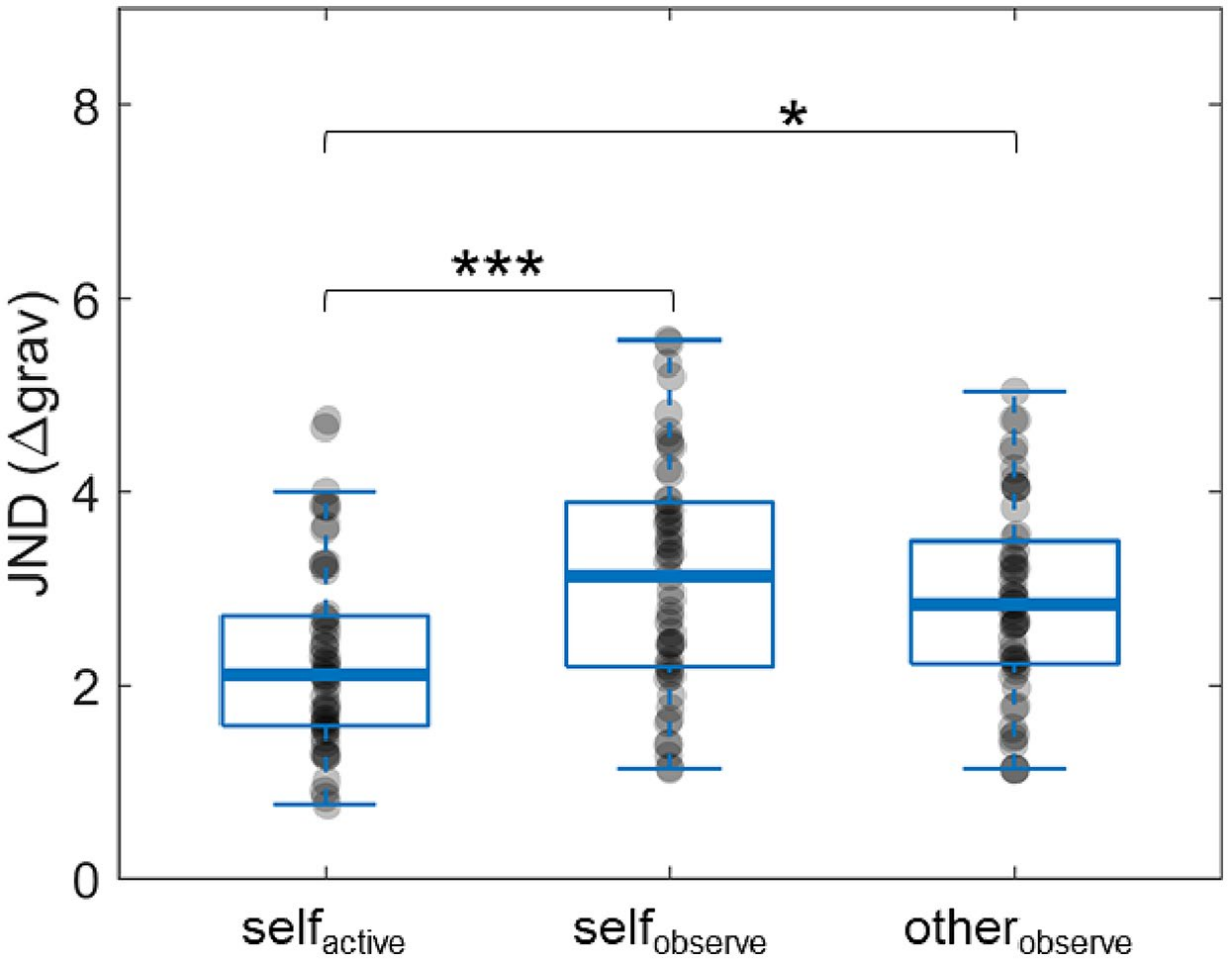
Within-condition just-noticeable difference (JND) results. Accuracy was measured by JND, defined here as the difference in gravity levels (Δ*grav*) where participants correctly identified the more difficult task in 80% of trials. Smaller JND indicates greater accuracy. *p < 0.05, ***p < 0.001. Note that in the plots the thick horizontal lines denote the median of the distribution.

We next compared the accuracies (JNDs) between the three experimental conditions (Figure 4). Participants were more accurate in *self_active_* trials (*Mean* = 2.29 ± 0.98) than in *self_observe_* (*Mean* = 3.11 ± 1.20) and *other_observe_* (*Mean* = 2.86 ± 1.01) trials. A repeated measures ANOVA revealed a statistically significant difference between conditions [*F*(2,100) = 19.791, *p* < 10^−7^, $\eta_{p}^{2}\;$ = 0.2836]. *Post hoc* pairwise comparisons with Bonferroni correction showed a significant difference between *self_active_* and *self_observe_* (*Mean Difference* = −0.82339, *SD* = 0.9557, *p* < 0.001, *d* = 0.8615) and between *self_active_* and *other_observe_* (*Mean Difference* = −0.57047, *SD* = 0.9423, *p* = 0.00022, *d* = 0.6053), but not between the two *observe* conditions (*Mean Difference* = 0.25293, *SD* = 0.9742, *p* = 0.209, *d* = 0.2661). Based on these results, we were able to reject the null hypothesis.

### 3.4 Hypothesis 4: no relationship between accuracy and bias

No correlation was found between *PSE_active vs observe_* and active/observe accuracy differences using either the preregistered (Pearson’s *r* = 0.08, *p* = 0.57, *d* = 0.1606) or the exploratory (*r* = 0.16, *p* = 0.27, *d* = 0.3243) model of accuracy driven bias (Supplementary Figures S3, S4). As hypothesized, the same analysis for self/other found no significant correlation (preregistered: *r* = −0.0008, *p* = 0.996, *d* = 0.0016; exploratory: *r* = −0.0016, *p* = 0.991, *d* = 0.0032). Thus, we found no evidence for a relationship between accuracy differences in the different task conditions and bias.

### 3.5 Exploratory results

**Figure 5:**
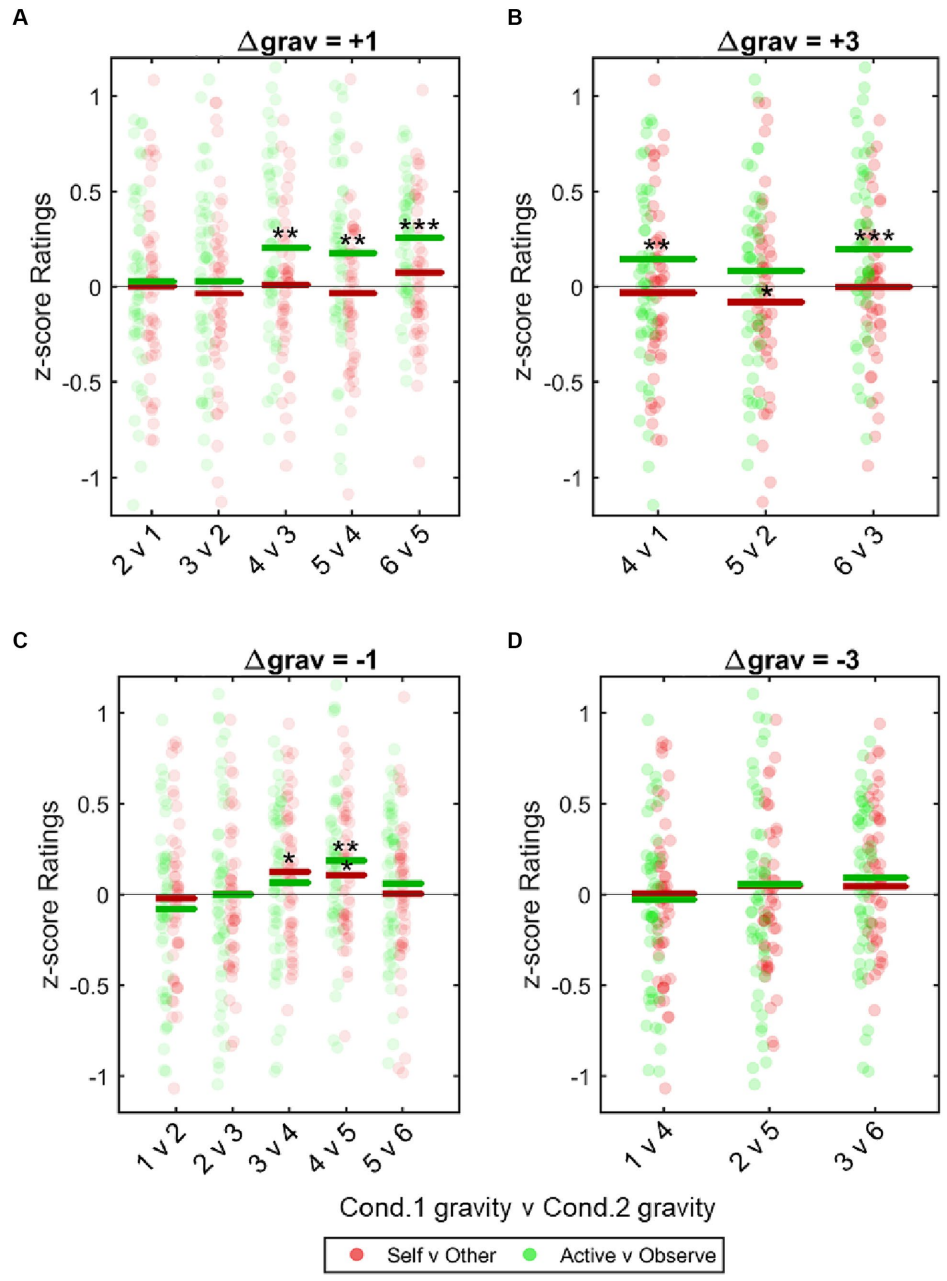
Effect of task difficulty on bias. Biases for each participant (expressed as *z*-scored differences in ratings) were calculated for all permutations for Δgrav = 3, 1, −1, −3. These were pooled across all participants and plotted to show bias as task difficulty increased. **(A, B)** *Post hoc* one sample *t*-tests show that when participants actively performed the more difficult task there was a significant bias for all trials where Condition (Cond.) 1 was ≥ gravity level 4 (apart from 5v2). **(C,D)** This effect was not seen when participants performed the easier of the two tasks. Data was analyzed with a repeated measures two-way ANOVA, with an interaction between bias type and task difficulty significantly affecting the variance and with *post hoc* one sample t-tests applied to each gravity level permutation. *p < 0.05, **p < 0.01, ***p < 0.001.

Our *a priori* hypothesis, that active/observe bias is driven by accuracy differences between active/observe conditions, was not supported. We next explored an alternative explanation, that bias was driven by sensory asymmetries between *active* and *observe* conditions, which would be intensified as the task difficulty increases. We used the continuous rating data to analyze and model the effect of task difficulty (Δ*grav*) on active/observe bias. An ANOVA (see Methods section 2.4.5) revealed that as the overall task difficulty increased, participants exhibited greater bias overestimating the difficulty of *active* task relative to *observe* trials (Table 4 and Figure 5)—albeit only when the *active* task was indeed more difficult than the observe task (Δ*grav* = +1, +3).

**Table 4:** Repeated measures ANOVA results for task difficulty and active/observe bias.

Δ*grav*	IV	DF	F	p	$\eta_{p}^{2}$
+1	*gl1*	(1,50)	17.65	0.0001	0.261
	Active	(1,50)	19.45	<0.0001	0.280
	gl1*Active	(1,50)	24.26	<0.0001	0.327
+3	*gl1*	(1,50)	4.31	0.043	0.079
	Active	(1,50)	27.34	<0.0001	0.354
	gl1*Active	(1,50)	26.45	<0.0001	0.346
−1	*gl1*	(1,50)	3.88	0.054	0.072
	Active	(1,50)	0.01	0.915	0.000
	gl1*Active	(1,50)	0.04	0.568	0.007
−3	*gl1*	(1,50)	2.53	0.118	0.048
	Active	(1,50)	0.05	0.823	0.001
	gl1*Active	(1,50)	0.14	0.713	0.003

Two independent variables (IV), gl1—the difficulty level of the Condition 1 task—and Active—a categorical parameter for whether there was an active/observe asymmetry—were assessed against group bias for the four Δgrav = gravity level 1 – gravity level 2.

Finally, we reran our mixed-effect modelling using the continuous rating data with an added fixed effect for overall task difficulty (see also Methods section 2.4.5). Both BIC and AIC analysis selected the Full model as the best fit for the data, indicating that the strength of the active/observe asymmetry depended on the task difficulty (Table 5).

**Table 5:** Exploratory model comparison assessing the interaction of task difficulty and bias.

Model	ΔAIC	ΔBIC	R^2^
Active + Self + *w*	118.99	56.44	0.600
Active + *w*	117.27	46.91	0.600
Self + *w*	129.36	59.00	0.600
Null	127.60	49.42	0.600
Full	0.00	0.00	0.603
Active + *g* * *w*	46.12	38.30	0.602
Active * *g* + *w*	68.44	60.62	0.602
Active + *g* + *w*	114.37	98.74	0.601
*g* + *w*	124.70	101.25	0.601

Using the subjective rating data, the preregistered models from Hypothesis 2—with categorical parameters for active/observe asymmetry (Active) or self/other asymmetry (Self) —were compared with models incorporating the gravity level of Condition 1 (g) as an interaction factor. The Full model included g as an interaction factor with both active/observe asymmetry and task presentation order. Adding this factor significantly improved the model fit according to both AIC and BIC assessment. All models contained a parameter that captured variance caused by task presentation order (w) which interacted with g in the Full model (Full model ~ active * g + g * w, Null model ~ w).

## 4 Discussion

This study examined the effect of attribution (self/other) and availability (active/observe) asymmetries on social comparison of effort judgements. Including a control condition where participants observed their own prerecorded activity made it possible to separate two proposed biases that are often undifferentiated within the literature. Our results showed that only active/observe asymmetries contributed to egocentric bias; self/other asymmetries had no significant effect. The bias measured in this study was small, with the psychometric modelling using the binary data indicating a mean bias of ~0.27 gravity levels, or 1.6% of a participants’ maximum effort as measured by *g_max_* (the exploratory analysis using continuous data estimated a more substantial ~0.6 gravity levels, or 3.6% of *g_max_*). However, this small effect size was expected, and was one of the primary motivations behind preregistering the study.

Our study provides insights into the underlying mechanism that drives egocentric bias. We predicted that greater access to sensory information while performing a task would lead to greater accuracy relative to simply observing a task, and that differences in accuracy between these two states would correlate with the degree of active/observe bias exhibited. This hypothesis was not supported by the results: While participants were significantly more accurate when comparing *active* tasks than *observe* tasks, there was no evidence of a correlation between differences in *active* and *observe* accuracy and the amount of bias. However, our exploratory analysis did support a correlation between the overall task difficulty and active/observe bias, albeit only in tasks where participants performed, not observed, the more difficult task. This correlation indicates that the active/observe bias is likely driven by sensory asymmetries inherent to the first-person perspective, for instance in the perceived level of exertion and fatigue, which are magnified as the task difficulty increases. Alternatively, the increased task difficulty may lead to the reallocation of cognitive resources away from other processes (such as those involved in empathy) needed to accurately observe, and may result in underestimation of observe trials. Furthermore, overestimating the difficulty of more effortful active tasks might be related to valuation biases stemming from sunk-cost effects of the extra energy the subjects already exerted on these trials. Combining our behavioral paradigm with the measurements of the biomarkers of exertion and fatigue (Lin et al., 2004; Gates and Dingwell, 2008) in future studies could establish a causal link between the sensory asymmetry and active/observe biases.

It is worth noting that the task difficulty in this study was intentionally relatively low. The study took an average of 4 h to complete; therefore, to avoid excessive discomfort or participant attrition, the task was set to a level around 60% of that used in previous iterations of the ball and ramp paradigm (Pooresmaeili et al., 2015; Rollwage et al., 2020). Given our results correlating increased bias with task difficulty, future studies incorporating greater task difficulty may find greater active/observe bias.

We found no bias indicating self/other asymmetries in judgements of task difficulty. However, this finding may be a direct result of the experiment’s incentive payments. Participants were explicitly rewarded for accuracy, which may have driven them to override any overt or motivated biases. While explicitly rewarding accuracy was intentional—we wanted to detect inherent egocentric biases without an external reinforcement—it is possible that a putative self/other bias was thwarted by our incentive structure. Another factor to consider is the self-relevance of the outcomes, which might have been too low to elicit self/other biases that are known to be stronger when maintaining a positive view of the self is essential (Campbell and Sedikides, 1999). Therefore, future studies could explore whether a different incentive structure (without rewarding the accuracy) or a stronger self-relevance of the outcomes (for instance by framing the existence of a link between the perceived task difficulty and a better social standing) could enhance the relative contribution of self/other biases. The possibility of a reduction in the attributional biases in the setting we tested is supported by the observation that participants were actively deploying metacognitive strategies to compensate for any inherent self/other bias. This was particularly evident in the *self_observe_* vs. *other_observe_* condition, where participants compared identical stimuli and yet rated the tasks attributed to their partner as significantly more difficult (Figure 3A). It is important to note, however, that this bias in the *self_observe_ vs other_observe_* condition did not affect the study’s ability to extract the unmotivated self/other bias, which was captured by the comparison between the main condition (*self_active_ vs other_observe_*) and the relevant control (*self_active_ vs self_observe_*).

Metacognitive strategy may also explain the exploratory result that task difficulty was correlated with greater bias only in trials where participants performed the more difficult task themselves (Figure 5). A participant countering their own egocentric bias to maximize their reward would focus on the correctness of their binary rating but not the accuracy of their continuous rating. This could lead participants to strategically apply a counterbias to trials that fell below a certain threshold of perceptual certainty. Given that our data indicated the participants overestimated *active* tasks, participants would, on this account, have greater perceptual certainty on trials where the *active* task was more difficult, while tasks where the *observe* task was more difficult would be perceived with less certainty and therefore be more likely to require metacognitive intervention. Further studies (e.g., using graded wagering; Moreira et al., 2018) are required to test this proposed mechanism.

Overall, this study underscores the importance of considering the information available to the self and others when biases in social comparison are assessed (Mussweiler, 2003). Our findings are in line with the previous work that took a similar approach (Ross and Sicoly, 1979; Pronin et al., 2004; Kruger et al., 2008; Moore and Small, 2011), but additionally, by dissociating different types of bias, they provide novel evidence that egocentric biases can occur in the absence of self vs. other asymmetries. These findings have important ramifications for how biases in social comparison can be understood and possibly modified through appropriate policies (Chambers and De Dreu, 2014). A self vs. other bias indicates a representational bias that has been reinforced over a lifespan (Palminteri et al., 2017) and is likely to resist change, whereas an active vs. observe bias, such as the one we found, is driven by information availability and therefore can be corrected readily by making information more symmetrical (e.g., through perspective taking; Zhou et al., 2013; Richardson et al., 2021). Together, these results support the notion that egocentric biases may indeed be a rational strategy to face the impoverished information that is available to agents when evaluating others, and can therefore be mitigated by enhancing their knowledge of the comparison group (Kruger et al., 2008; Chambers and De Dreu, 2014). An exciting direction for future studies would be to explore whether providing specific information on the level of effort experienced by others could enhance people’s prosocial choices to take on an effortful action that benefits others (Lockwood et al., 2017), especially in transparent face-to-face settings that might facilitate cooperative tendencies (Moeller et al., 2023).

There are, however, some notes of caution while considering these results. This study focused solely on bias inherent to the egocentric perspective in judging task difficulty. As such, a range of potential biases driven by self/other asymmetries were not addressed. Well documented biases that are better described by this asymmetry (e.g., the endowment effect; Kahneman et al., 1991) were necessarily excluded by our paradigm. Furthermore, the social aspect of our experiment paired strangers from relatively homogenous demographics, and as such did not explore potential attributional biases produced by perceived differences between oneself and others (Ashkanasy, 1997; Green and McClearn, 2010). Moreover, our task was not a joint task towards a common goal that might elicit a stronger self-serving bias, as often considered in the egocentric bias literature (Ross and Sicoly, 1979). Nevertheless, estimating own and others’ effort spent on a similar task is highly relevant for many real-life social comparisons (Kruger et al., 2008). A question might arise regarding the ecological validity of the task we used and how generalizable the findings from a computer-based laboratory task are to real-world settings. We note that working side-by-side on a computer is a common experience in the modern world, and indeed, it has been previously shown that the effects transpiring in such tasks were related to participants’ real-world views (Rollwage et al., 2020). Given these considerations, this study makes no comment on the existence or prevalence of attributional self/other biases in general, but rather aims to better characterize the bias inherent in egocentric perception.

## 5 Conclusion

Our study featured a novel paradigm that allowed us to dissociate between egocentric bias driven by *active/observe* and by *self/other* asymmetries. We found that participants perceived tasks they performed to be harder than tasks they observed, but found no bias correlating with whether the participant or their partner performed the task. Furthermore, exploratory analysis suggested that as the effortful task became more difficult, participants exhibited a greater bias towards overestimating *active* tasks. Together, these results suggest that egocentric biases in effort judgements may occur in the absence of self/other asymmetries, but they do rely on the active/observe asymmetries to emerge.

## Data availability statement

The raw data supporting the conclusions of this article will be made available by the authors, without undue reservation.

## Ethics statement

The studies involving humans were approved by Freie Universität Berlin Department of Educational Science and Psychology Ethics Commission. The studies were conducted in accordance with the local legislation and institutional requirements. The participants provided their written informed consent to participate in this study. Written informed consent was obtained from the individual(s) for the publication of any potentially identifiable images or data included in this article.

## Author contributions

CS: Conceptualization, Data curation, Formal analysis, Investigation, Methodology, Project administration, Validation, Visualization, Writing – original draft, Writing – review & editing. IK: Conceptualization, Funding acquisition, Supervision, Visualization, Writing – review & editing. AP: Conceptualization, Funding acquisition, Methodology, Resources, Supervision, Validation, Writing – original draft, Writing – review & editing.

## Footnotes

^1^ https://osf.io/6pwvf/?view_only=fcbbd52a25ca44e9989254a97748868d
