# Supplementary material for: The contribution of sensory information asymmetry and bias of attribution to egocentric tendencies in effort comparison tasks
Source: Front Psychol. 2024 Apr 4;15:1304372. doi: 10.3389/fpsyg.2024.1304372 (PMC11025643; doi:10.3389/fpsyg.2024.1304372)
Supplement: Supplementary file 1 [file Presentation_1.pdf]

Supplementary Material: The contribution of sensory information asymmetry and bias of attribution to egocentric tendencies in effort comparison tasks

Caedyn Stinson, Igor Kagan, Arezoo Pooresmaeili

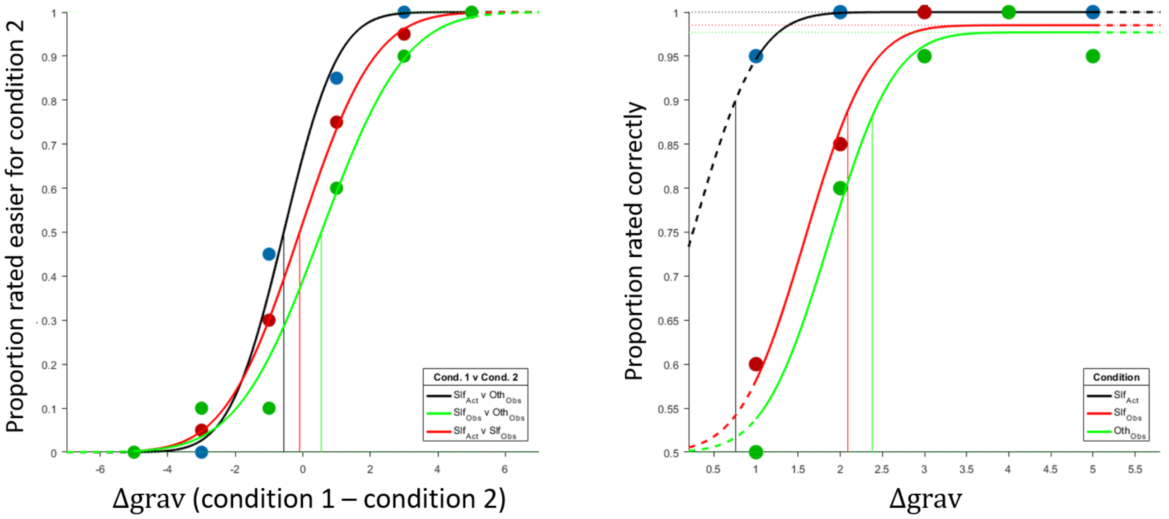

Figure S1. *Psychometric curves of a typical participant*. For each participant PSE and JND were estimated based on psychometric functions fit to the data of between-condition (left) or within-condition (right) estimations of task difficulty (parametrised by differences in gravity level, i.e., Δgrav). The average PSEs and JNDs across participants estimated from these psychometric functions are shown in Figure 3 and Figure 4 of the main text.

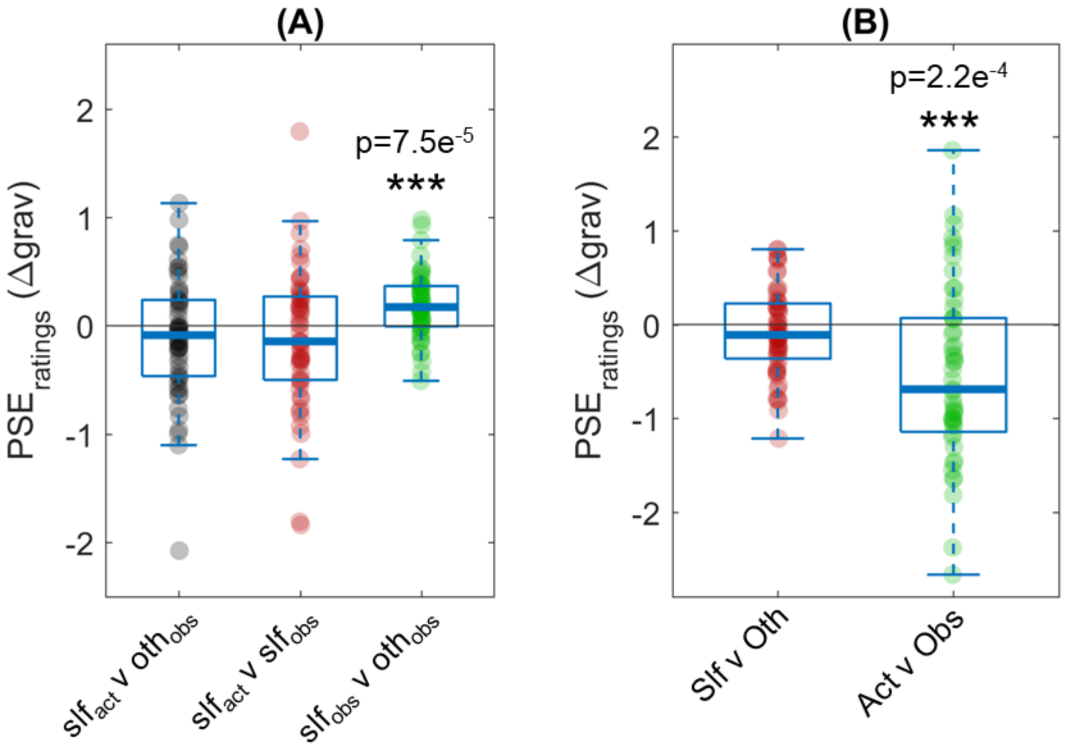

Figure S2. *Continuous Rating Based Point of Subjective Equality ($PSE_{ratings}$) Analysis of Bias.* Both panels plot the $PSE_{ratings}$, with negative values indicating an overestimation of the difficulty of the condition listed first (i.e. Condition 1). (A) shows biases in ratings between the three conditions, with no bias detected at a group level for slf_{act} vs oth_{obs} ($t(50) = -1.33$, $p=0.190$) or slf_{act} vs slf_{obs} ($t(50) = -1.33$, $p = 0.191$). Participants significantly underestimated the difficulty of their own trials in slf_{obs} vs oth_{obs} comparisons ($t(50) = 4.31$, $p <0.0001$). (B) displays attribution bias (Slf vs Oth) and sensory asymmetry bias (Act vs Obs) for all participants. At a group level, only sensory asymmetry bias was detected ($t(50) = -3.99$, $p = 0.0002$) with no differences found for attribution bias ($t(50) = - 1.21$, $p = 0.234$).

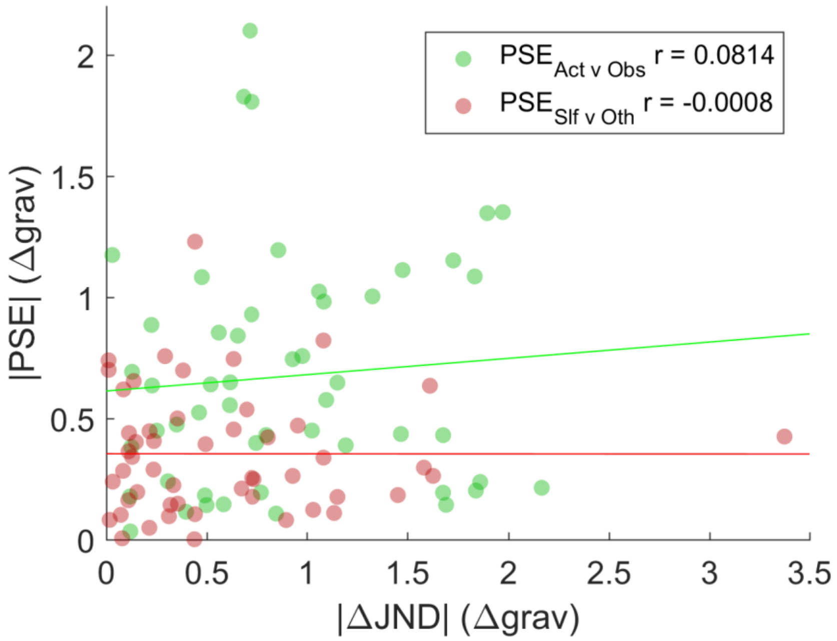

Figure S3. *Analysis of the correlation between relative accuracy and bias (pre-registered analysis)*. The absolute value of participant's bias (|PSE|) is plotted against the absolute differences in their accuracy between either *Active* and *Observe* tasks (green) or *Self* and *Other* tasks (red). The correlation is given by *Pearson's r*, with neither correlation significant (*Active vs Observe* $p = 0.5702$, *Self vs Other* $p = 0.9958$).

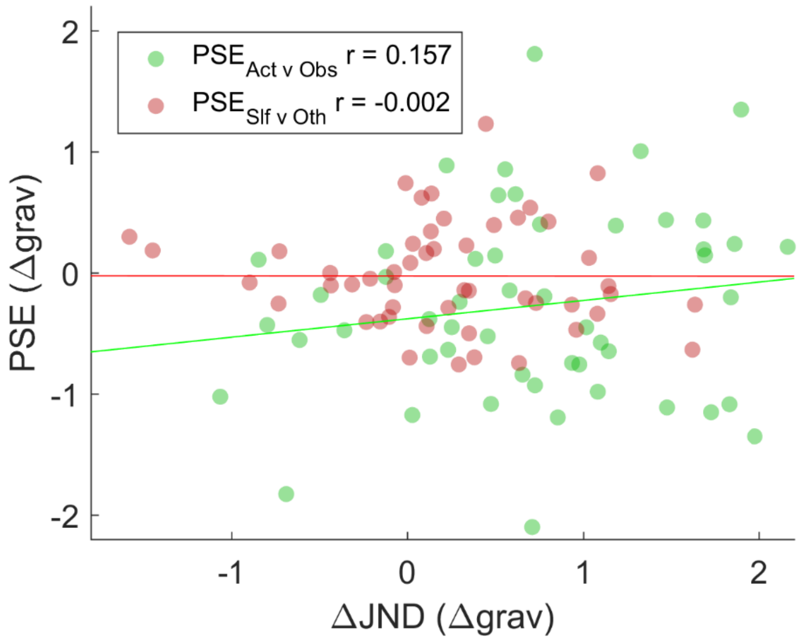

Figure S4. *Analysis of the correlation between relative accuracy and bias (exploratory analysis)*. The signed value of participant's bias (PSE) is plotted against the differences in their accuracy between either *Active* and *Observe* tasks (green) or *Self* and *Other* tasks (red). The correlation is given by *Pearson's r*, with neither correlation significant (*Active vs Observe p* = 0.2706, *Self vs Other p* = 0.9914).

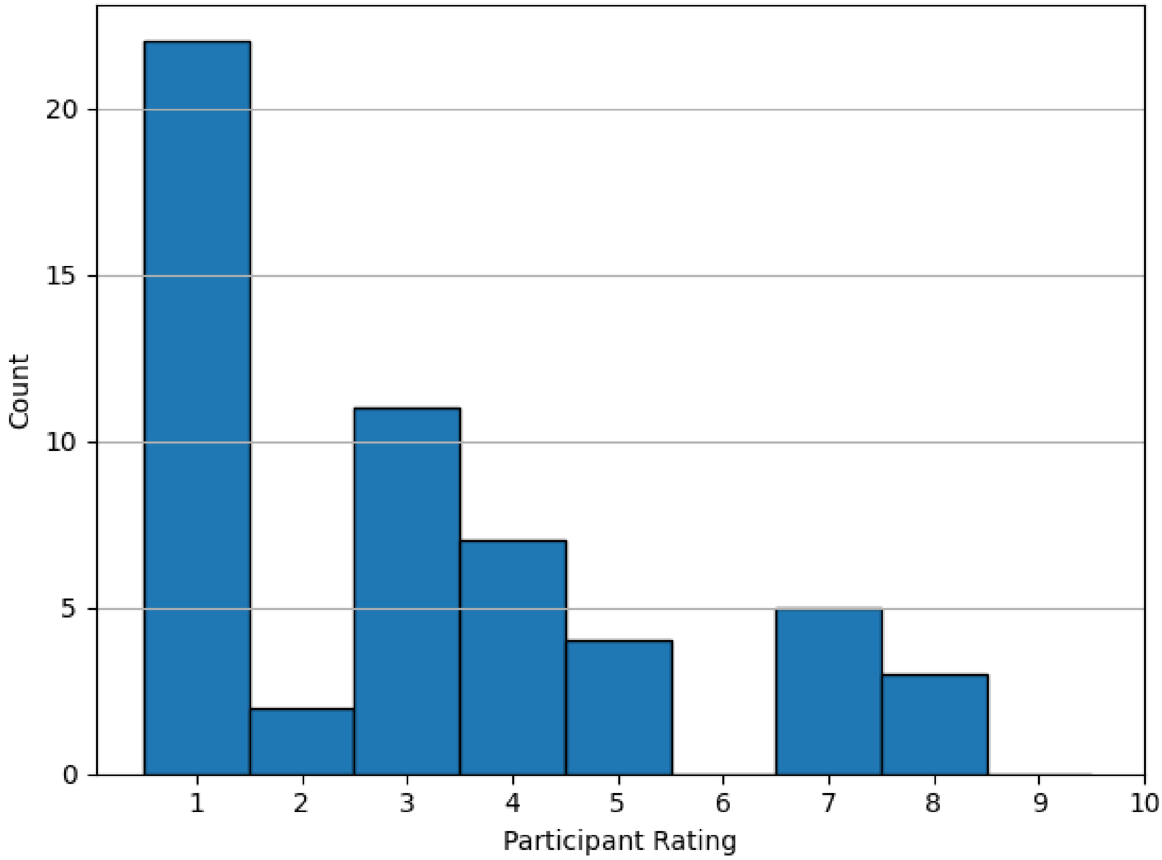

Figure S5. *Participant responses to manipulation detection question.* After learning of our experimental manipulation, participants were asked the question: "On a scale of 1–10, with 1 being not at all, and 10 being completely, to what degree did you suspect the other participant's activity wasn't their own?" Our pre-registered exclusion criteria required a participant to be excluded if they responded with 10, therefore no participants were excluded following this question.

Table S1

Attribution Bias and Sensory Asymmetry Bias Model Comparison with Rating Data

Model	ΔAIC	ΔBIC	Log Likelihood	R^2 (adjusted)
$PSE_{ratings}$ full	1.72	9.54	-18041	0.3789
$PSE_{ratings}$ active	0	0	-18042	0.3823
$PSE_{ratings}$ self	37.51	37.51	-18060	0.3714
$PSE_{ratings}$ null	44.15	36.34	-18065	0.3758

Note. Models featuring both attribution bias and sensory asymmetry bias ($PSE_{ratings}$ full) or only one bias ($PSE_{ratings}$ self and $PSE_{ratings}$ active) were fit with the between-condition PSE data. In contrast to the PSE results, model comparisons using both AIC and BIC were in agreement that the model with a lone bias parameter for *Active vs Observe* best explained the data.
